# Risk factors of infective endocarditis in persons who inject drugs

**DOI:** 10.1186/s12954-020-00378-z

**Published:** 2020-06-05

**Authors:** Meera Shah, Ryan Wong, Laura Ball, Klajdi Puka, Charlie Tan, Esfandiar Shojaei, Sharon Koivu, Michael Silverman

**Affiliations:** 1 Schulich School of Medicine & Dentistry, Western University, London, ON Canada; 2 Western University, London, ON Canada; 3 Department of Epidemiology and Biostatistics, Western University, London, ON Canada; 4 Division of Infectious Diseases, St. Joseph’s Health Care, London Health Sciences Centre, London, ON Canada; 5 Department of Family Practice, Western University, London, ON Canada; 6 Division of Infectious Diseases, Department of Medicine, Schulich Medicine & Dentistry, Room B3-414 268 Grosvenor Street, London, ON N6A 4V2 Canada

**Keywords:** Injection drug use, Infective endocarditis, HIV, Hepatitis C, Opioid precedence, Opioid epidemic, Hydromorphone, Female PWID

## Abstract

**Background:**

The rising incidence of infective endocarditis (IE) among people who inject drugs (PWID) has been a major concern across North America. The coincident rise in IE and change of drug preference to hydromorphone controlled-release (CR) among our PWID population in London, Ontario intrigued us to study the details of injection practices leading to IE, which have not been well characterized in literature.

**Methods:**

A case–control study, using one-on-one interviews to understand risk factors and injection practices associated with IE among PWID was conducted. Eligible participants included those who had injected drugs within the last 3 months, were > 18  years old and either never had or were currently admitted for an IE episode. Cases were recruited from the tertiary care centers and controls without IE were recruited from outpatient clinics and addiction clinics in London, Ontario.

**Results:**

Thirty three cases (PWID IE+) and 102 controls (PWID but IE-) were interviewed. Multivariable logistic regressions showed that the odds of having IE were 4.65 times higher among females (95% CI 1.85, 12.28; *p* = 0.001) and 5.76 times higher among PWID who did not use clean injection equipment from the provincial distribution networks (95% CI 2.37, 14.91; *p* < 0.001). Injecting into multiple sites and heating hydromorphone-CR prior to injection were not found to be significantly associated with IE. Hydromorphone-CR was the most commonly injected drug in both groups (90.9% cases; 81.4% controls; *p* = 0.197).

**Discussion:**

Our study highlights the importance of distributing clean injection materials for IE prevention. Furthermore, our study showcases that females are at higher risk of IE, which is contrary to the reported literature. Gender differences in injection techniques, which may place women at higher risk of IE, require further study. We suspect that the very high prevalence of hydromorphone-CR use made our sample size too small to identify a significant association between its use and IE, which has been established in the literature.

## Background

There has been a rising incidence of infective endocarditis (IE) among people who inject drugs (PWID), paralleling the opioid epidemic in North America [1–6]. This infectious complication of intravenous drug use (IVDU) is associated with significant morbidity and mortality requiring extensive and costly [1, 2] multidisciplinary care [7]. PWID are at risk of IE due to comorbidities of HIV, hepatitis C, skin and soft tissue infections, non-sterile injection practices, and the reuse of injection drug preparation equipment (IDPE) [2, 4, 8–12].

**Fig. 1:**
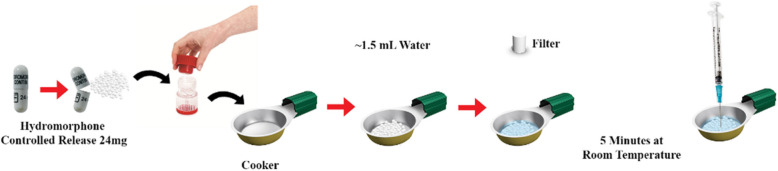
Process of preparation and injection of hydromorphone controlled-release capsule for injection drug use. Storage of the used cooker and filter for use of residual hydromorphone is almost very commonly performed and leads to bacterial contamination [12, 14]. Heating the cooker with a cigarette lighter prior to use reduces bacterial burden [12]

In June of 2016, a public health emergency was declared in London, ON, Canada following an increase in IE [13] and injection-related HIV [14]. We identified an association between increasing IVDU-associated IE hospitalization and increasing prescriptions of hydromorphone across Ontario [4]. Prescription rates for hydromorphone controlled-release (CR; Hydromorph Contin ®, Purdue Pharma, Pickering, Ontario) in London are in the top quartile in Ontario [4, 13, 15]. Furthermore, studies have shown that the misuse of prescribed opiates such as hydromorphone-CR amplifies the risk of infections due to the nature of how these substances are prepared for injection [11, 16–18]. Hydromorphone-CR is difficult to dissolve in solution, often requiring PWID to use unsterile methods to prepare injectates. The components of hydromorphone-CR capsules that provide the controlled release, increase survival of HIV [19] and *Staphylococcus aureus* [12]*,* which cause the vast majority of IE cases [7]. Moreover, residual drug remains in cookers and filters after an initial injection, allowing PWID to resolubilize the remaining drug and conduct multiple injections (Fig. 1) [11, 14, 16, 19, 20]. This high-risk practice of multiple injections involves keeping, sharing, and reusing injection drug preparation equipment (IDPE), which increases infections among PWID [14, 16, 20]. However, studies have also found that heating the injectate before injection of hydromorphone-CR can significantly reduce the inoculum of bacteria causing IE [12].

With the coincident rise in IE and change of drug preference to hydromorphone-CR among PWID in London, Ontario, this study aimed to identify demographic variables and injection practices that pose a risk for IE. We hypothesized that using hydromorphone-CR would increase the risk of IE. Secondly, we hypothesized that being a male, injecting into multiple sites, using non-sterile equipment, and failing to heat hydromorphone-CR injectates would be further risk factors for IE. As a secondary goal, we also sought to explore demographic variables and injection practices of PWID in London to generate hypotheses for further studies.

## Methods

### Design/setting/participants

We conducted a case–control study where persons who inject drugs (PWID) ≥ 18 years were eligible for participation. To be classified as a PWID, participants had to self-report injection drug use within the last 3 months. Our cases were PWID with “Definite IE”, based on the Modified Duke Criteria [21]. Cases were recruited from the three tertiary care centers in London that provide all inpatient care for patients with endocarditis. Outpatients being followed up for recent IE (within 6 weeks) were also recruited as cases through the outpatient Infectious Disease clinics covering London. Our controls were PWID with no history of IE episodes; these participants were recruited from addiction and outpatient clinics commonly serving PWID in London, Ontario. Recruitment for controls was conducted at Addiction Services of Thames Valley, Regional HIV/AIDS Connection, St Joseph’s Health Care Infectious Disease clinics and the London Intercommunity Health Center. This choice of sites allowed us to observe a diverse subset of participants with varied injection practices. Sampling from the Regional HIV/AIDS and Addiction Services of Thames Valley allowed us to observe participants who had access to counselling, whereas sampling from St Joseph’s Healthcare and London Intercommunity Health Center allowed us to observe participants who had riskier injection practices complicated with infections other than IE. We recruited participants from August 11, 2016 to July 27, 2018.

Anonymous interviews were conducted with a questionnaire querying demographic data, medical history pertaining to current and previous IE episodes (if any) and other infectious complications, and history of intravenous drug use in the past 3 months and over one’s lifetime. Each participant was then assigned an identification number. Patient medical records were consulted by Infectious Disease physicians to verify definite cases of IE. Study data were digitized and managed using REDCap electronic data capture tools hosted at Lawson Health Research Institute [22]. The study protocol was approved by an institutional board review (Health Sciences Research Ethics Board, Western University). Written informed consent was obtained from all participants. Participants were compensated for their time with a Canadian $10 Tim Horton’s coffee shop gift card. Reporting of all aspects of this study adhered to the Strengthening the Reporting of Observational Studies in Epidemiology (STROBE) reporting guideline for cohort studies [23].

### Statistical analysis

Analyses were completed using R 3.6.2 [24]. Demographic and clinical characteristics of cases (PWID IE+) and controls (PWID IE–) were evaluated through independent samples t-tests (for continuous variables) and chi-square or Fisher’s exact test (for categorical variables). To evaluate our first hypothesis, we used chi-square analysis to compare the proportion of cases (IE+) and controls (IE−) who utilized hydromorphone-CR. To evaluate our second hypothesis, we compiled two multivariable logistic regression models to evaluate variables we hypothesized would increase the risk of IE. One model included gender, use of government-distributed IDPE (Stericup) and injection into multiple sites among all participants. The other model included the previous variables and additionally, whether PWID do not heat their hydromorphone-CR; this model was completed among hydromorphone-CR users because the additional variable only applied to these individuals. Although reuse of hydromorphone-CR for a second wash is a known risk factor of IE, it was not placed in these models given our small sample size and the ubiquity of this practice our cohort; similarly, use of hydromorphone-CR was also not entered into the multivariable model.

## Results

One hundred and forty-one interviews were conducted, with 135 individuals being included in the final study. Of the 34 case interviews conducted, 1/34 (3%) was excluded from the final study as this individual did not have a definite episode of IE; thus, 33/34 (97%) were included in the final study. Our cases primarily had tricuspid-valve endocarditis and the most common organism causing IE was methicillin-sensitive *Staphylococcus aureus* (Table 1). Of the 107 control interviews conducted, 4/107 (4%) were excluded as they had stopped injecting drugs for more than 3 months prior to interview. Moreover, 1/107 (1%) was excluded due to inconsistent answers being provided between the different sections of the interview. In finality, 102/107 (95%) controls were included in the final study. The demographic characteristics of cases and controls are presented in Table 2 and discussed below.

**Table 1:** Microbial etiology and site of endocardial involvement in PWID with IE

Endocarditis characteristics	
Variable (%)	***n*= 33**
**Organism**	
MSSA	23 (69.7%)
MRSA	5 (15.1%)
Enterococci	2(6.0%)
Other	3(9.1%)
**Infected valve**	
Tricuspid	24 (72.7%)
Pulmonic	0 (0%)
Mitral	6 (18.1%)
Aortic	2 (6.0%)
Unknown	1(3%)

**Table 2:** Demographic characteristics and previous complications of PWID stratified by endocarditis history

Variable (%)	Control (***n*** = 102; %)	Case (***n*** = 33; %)	*p* value
**Demographical characteristics**			
Female	21/102 (20.6)	16/33 (48.5)	0.002
Mean age (years, std dev)	40.0 (11.0)	35.5 (8.4)	0.034
Caucasian	74/102 (72.5)	28/33 (84.8)	0.17
Stable housing	45/102 (41.6)	12/33 (53.1)	0.35
Completion of secondary or post-secondary	31/92 (33.7)	19/31 (61.3)	0.010
Employed or seasonally employed	6/100 (6.0)	3/30 (10.0)	0.43
HIV	30/102 (29.4)	8/33 (24.2)	0.57
Hepatitis C	25/102 (24.5)	7/33 (21.2)	0.69
**Previous complications**			
Cellulitis	67/101 (66.3)	20/32 (60.6)	0.54
Cotton fever	84/101 (83.2)	24/32 (75.0)	0.30
Osteomyelitis	7/101 (6.9)	3/32 (9.4)	0.70
Pneumonia	39/101 (38.6)	17/31 (54.8)	0.11

*std dev* standard deviation

With respect to our first hypothesis, the most commonly injected drug in both groups was hydromorphone-CR; however, hydromorphone-CR use was not significantly different between cases (91%) and controls (81%; *p* = 0.20). Cases and controls injected a wide range of drugs (Table 3); hydromorphone-immediate release tablets was the second most commonly injected drug. Furthermore, there was a trend towards crystal methamphetamine being injected by control participants more than case participants (54.4% cases; 78.4% controls; *p* = 0.07). Injection of fentanyl tablets or patches was sparse among PWID in London at this time.

**Table 3:** Summary of PWID intravenous drug use stratified by endocarditis history

Variable (%)	Control (***n*** = 102; %)	Case (***n*** = 33; %)	*p* value
**Drug injected**			
Oxycodone hydrochloride tablets (Oxycontin)	43 (42.2)	10 (30.3)	0.22
Hydromorphone controlled-release capsules (Hydromorph contin)	83 (81.4)	30 (90.9)	0.20
Crystal methamphetamine	80 (78.4)	18 (54.5)	0.07
Hydromorphone controlled-release tablets (Dilaudid)	76 (74.5)	21 (63.6)	0.23
Bupropion (Wellbutrin)	0 (0)	1 (3)	0.24
Methylphenidate (Ritalin)	18 (17.6)	4 (12.1)	0.60
Cocaine	16 (15.7)	3 (9.1)	0.41
Crack	8 (7.8)	0	0.20
Fentanyl patch	1 (1.0)	3 (9.1)	0.45
Fentanyl tablet	4 (3.9)	1 (3.0)	1.00
Heroin	14 (13.7)	4 (12.1)	1.00
**Injection location most frequently used**			0.012^a^
Arm	62 (64.6)	12 (48.0)	
Hand	3 (3.1)	2 (8.0)	
Neck	12 (12.5)	2 (8.0)	
Leg*	6 (6.2)	1 (4.0)	
Foot	1 (1.0)	0 (0.0)	
Multiple sites**	12 (12.5)	8 (32.0)	
Injection location used in the past 3 months			0.38^a^
Hand***	13 (14.8)	1 (3.4)	
Lower leg	1 (1.1)	1 (3.4)	
Feet	1 (1.1)	0 (0.0)	
Neck	8 (9.1)	3 (10.3)	
Multiple sites**	65 (73.9)	24 (82.8)	

* Leg includes injection into the femoral vein and calf

** Many different sites used

*** Note all hand patients used arm in the last 3 months

^a^ Fisher’s exact

With respect to our second hypothesis, we evaluated the association of IE and sex, government-dispensed IDPE, location of injection, and heating of hydromorphone-CR. Multivariable logistic regression analyses found that being female and not using government-dispensed IDPE (Stericups) were independently associated with having IE in unadjusted and adjusted models (Tables 4 and 5); injecting in multiple sites and always heating hydromorphone-CR were not significantly associated with IE. In the adjusted model, among all participants, females had 4.65 times higher odds of having IE (95%CI 1.85, 12.28; *p* = 0.001), and those who did not use government-distributed IDPE had 5.76 times higher odds of having IE (95% CI 2.37, 14.91; *p* < 0.001).

**Table 4:** Multivariable model of risk factors for infective endocarditis, among all participants

	Unadjusted OR (95% CI)	***p*** value	Adjusted OR (95% CI)	***p*** value
Sex, female	3.85 (1.68, 8.96)	0.002	4.65 (1.85, 12.28)	0.001
Does not use Stericup*	4.96 (2.18, 11.59)	< 0.001	5.76 (2.37, 14.91)	< 0.001
Most frequently injects at multiple sites	2.29 (0.83, 6.07)	0.1	1.67 (0.53, 4.97)	0.36

*OR* odds ratio, *CI* confidence interval

*Clean equipment from harm reduction services

**Table 5:** Multivariable model of risk factors for infective endocarditis, among hydromorphone controlled-release (CR) users

	Unadjusted OR (95% CI)	***p*** value	Adjusted OR (95% CI)	***p*** value
Sex, female	3.94 (1.64, 9.67)	0.002	4.20 (1.56, 11.96)	0.005
Does not use Stericup*	5.92 (2.46, 14.86)	< 0.001	6.77 (2.61, 18.96)	<.001
Most frequently injects at multiple sites	2.30 (0.81, 6.30)	0.11	1.89 (0.57, 6.04)	0.29
Never heats hydromorphone-CR	1.60 (0.66, 4.21)	0.32	1.57 (0.57, 4.71)	0.4

*OR* odds ratio, *CI* confidence interval

*Clean equipment from harm reduction services

Lastly, we also sought to explore the demographic variables and injection practices of PWID in London to generate hypotheses for further studies. The demographic characteristics of the two cohorts are shown in Table 2. Cases also more commonly had completed secondary or post-secondary education (did not have incomplete degrees) compared to controls (61.3% cases; 33.7% controls; *p* = 0.01). Controls and cases were similar in age, race, housing status, concurrent HIV and hepatitis C infections, and past complications.

Injection location data (Table 3) showed that cases and controls differed in the location they frequently injected (Fishers’ exact test, *p* = 0.012); this difference was driven by the higher frequency of multi-site (32% vs 13%) among cases compared to controls.

The injection preparation techniques and behaviors of cases and controls are highlighted in Table 6. Both cases and controls used cookers to prepare drugs for injection at a rate of 50–60%. Controls were more likely to use a Stericup, which is distributed in the IDPE kits by the provincial government (42.4% cases; 84.2% controls; *p* < 0.001) to prepare drugs. Furthermore, PWID with IE were more likely to use cooker types that were not listed in our surveys (Stericup, spoon, glass bottle, or shot glass) than controls (48.5% cases; 11.8% controls; *p* < 0.001).

**Table 6:** Summary of PWID intravenous drug use behaviors stratified by endocarditis history

Variable (%)	Control (%)	Case (%)	*p* value
**Cooker use**			
Uses cooker with all drugs	60 /101 (59.4)	17/33 (51.5)	0.43
Uses a Stericup*	84/102 (84.2)	14/33 (42.4)	**< 0.001**
Unknown** cooker type	12/102 (11.8)	16/33 (48.5)	**< 0.001**
**Heating**			
Heats drugs in cookers (any drug)	70/101 (68.6)	28/33 (84.2)	*0.07*
Always heats hydromorphone-CR	31/82 (37.8)	8/30 (26.7)	0.38
Always or sometimes heats hydromorphone-CR and its subsequent washes	43/82 (52.4)	14/30 (46.7)	
**Preparation**			
Soaks drugs before injection	45/99 (45.5)	14/30 (46.7)	0.91
Reuse of hydromorphone-CR for a second wash	76/102 (74.5)	29/33 (87.9)	0.11
**Filter use**			
Cellulose filter used	97/101 (96.0)	31/32 (96.9)	0.83
Cigarette filter used	84/101 (83.2)	24/32 (75.0)	0.30
Reuses filters	65/102 (63.7)	20/33 (60.6)	0.75
Store filter in cooker	26/102 (25.5)	21/33 (36.4)	0.23
Store filter in pockets/body temperature	31/102 (30.4)	9/33 (28.1)	0.81
Shares filters	18/100 (18.0)	5/32 (15.6)	0.90
**Cleaning**			
Cleans skin with alcohol swab before injection	69/74 (93.2)	18/20 (90.0)	0.64
Always cleans with alcohol swab before injection	33/74 (44.6)	5/20 (25.0)	0.13
Always cleans with alcohol swab and heats drug before injection	49/74 (66.2)	10/20 (50.0)	0.29
**Crushers**			
Pill grinder	16/102 (15.7)	4/33 (12.1)	`0.78
Lighter	60/102 (58.8)	12/33 (36.4)	***0.025***
Sharing crushers	36/97 (37.1)	14/29 (48.3)	0.28
**Needles**			
Reuse of needles	76/102 (74.5)	23/33 (69.7)	0.59
Sharing of needles	24/102 (23.5)	4/33 (12.1)	0.16
Reuse of syringe barrel	68/102 (66.7)	20/33 (60.6)	0.52

* Clean equipment from harm reduction services

** Refers to cookers that were not Stericups, spoons, glass bottles or shot glasses

A cellulose filter was commonly used by both groups (96%). Both groups indicated reusing their filters at 63.7% controls and 60.6% cases for multiple injections. Additionally, both groups stored their filters in cookers or pockets and/or at body temperature in similar proportions. Many participants (18% controls and 15.6% cases) shared their filters.

In terms of reusing the hydromorphone-CR capsule for multiple injections (‘washes’), both cases and controls conducted second washes frequently (87.9% cases; 74.5% controls; *p* = 0.11).

We did not find that heating drugs were protective; however, we did find that controls used lighters as crushers for drug preparation significantly more than cases (58.8% vs 36.4%; *p* = 0.025), suggesting that controls may have greater accessibility to a heating source.

While there were no significant differences between cohorts in the frequencies of needle and syringe sharing or reuse, the rates of reuse are high with 74.5% of controls and 69.7% of cases reporting reuse of needles. Furthermore, the rate of reuse for syringe barrels also remains high with 66.7% of controls and 60.6% of cases reporting reuse.

## Discussion

Understanding the risk factors associated with IE in PWID is important in developing harm reduction strategies. We hypothesized that the use of hydromorphone-CR would be a risk for IE among PWID; however, we did not find a significant increase in hydromorphone-CR use in IE patients vs controls (91% vs 84%). In contrast, our previous work has demonstrated evidence of such a relationship. Our population-wide study in Ontario with over 60,000 PWID showed a 3.3-fold higher risk of acquiring IE within 120 days when prescribed hydromorphone-CR compared with other opioids (*p* < 0.0001) [25]. Moreover, we have also shown that drug excipients within hydromorphone-CR preserve *S. aureus* survival in vitro [12]. This was not the case for immediate release hydromorphone or controlled-release Oxycodone [12]. Furthermore, we found that the injectate obtained from aspirating from equipment previously used to inject hydromorphone-CR was contaminated with *S. aureus* in 14% of cases, and thus, injection of this drug would commonly be associated with bacteremia [12]. We suspect that the very high prevalence of hydromorphone-CR use in both cases and controls led to a lack of power to identify a difference in use between the two groups in this study.

There has been very little data assessing the detailed injection practices associated with developing IE. The literature primarily studies the clinical and epidemiological characteristics of PWID developing IE [3, 5, 7, 26, 27]. Some studies assessing injection practices of PWID are in relation to the development of skin and soft tissue infections [28] or infections in general [14, 20, 29]. To our knowledge, this is the largest study (*n* = 33) showcasing detailed survey data regarding injection practices of PWID with IE. Understanding PWID-IE risk factors are of importance to inform public health authorities in the development of harm reduction strategies reducing infections in this at-risk population. Our one-on-one surveys have allowed for the collection of comprehensive quantitative and qualitative data to thoroughly understand injection practices and behaviors of PWID in our region to elucidate the etiology of our high IE rates.

Previous studies suggested that IE in PWID was more frequently seen in males, younger patients, and those with concurrent HIV infections [3, 8, 9]. However, our cases and controls had similar ages, concurrent HIV, and HCV infections. Nearly a quarter of our cases and controls had HIV; the high incidence of HIV in this population is likely related to our co-existent local HIV epidemic [19]. Hepatitis C rates were based on self-report, and a lack of awareness of status may have led to lower than expected rates in both cases and controls.

Unexpectedly, being a female PWID was a risk factor for IE in our population (OR 4.65; 95% 1.85–12.28). Wurcel et al. also showed a greater parity in PWID-IE distribution by sex (female = 53%) between the ages of 15–34 over a 13-year review of IE hospitalizations in the USA [5]. We suspect that gender differences may exist with regards to injection technique. Women are more likely to have sex partners that initiate them into injection practices and are more likely to share IPDE than men [30, 31]. Women can be identified sub-populations for targeted harm reduction, and in particular, interventions should account for intimate partner dynamics concerning high-risk practices [32]. Furthermore, female anatomy increases the difficulty of IVDU. We hypothesize that women have smaller veins, which may be difficult to visualize, often requiring increased manipulation during injections. This inability to find an adequate injection site with smaller veins can promote the usage of larger, more accessible central veins like the internal jugular, which further increases risks of infection. Additionally, local surveys in our region from the Middlesex-London Health Unit found that female PWID in London were more likely to borrow and share their IDPE [33]. It was also anecdotally noted, through our other project in progress, that women were less likely to access supervised injection sites, leading to unsafe injection practices that place them at risk of IE.

Usage of provincial-distributed IDPE, i.e., the Stericup, for mixing drugs was found to be protective against IE. PWID who are more likely to use equipment from needle exchange programs are also more likely to be exposed to education on safe injection practices and consistently use sterile equipment. PWID with IE were also more likely to use objects for mixing and heating drugs that were not distributed through IDPE kits or commonly listed in our interview questions. This suggests that cases might be injecting in severe withdrawal states, where concern for safe practices do not take precedence over the need to use. Additionally, the increased use of a lighter may be suggestive that controls are using drugs that require heating such as heroin, crystal methamphetamine, and cocaine, and these may reflect a lower risk of using hydromorphone-CR. However, we did not see a significant protective effect of always heating hydromorphone-CR preparations (OR 1.57; 95% CI 0.57, 4.71). We feel this may be due to the variations in practice that PWID follow. It is likely that PWID heat their drugs depending on the circumstance (state of withdrawal, supervised site, environmental factors, etc.), and this information was not captured in our line of questioning. We asked participants to choose a definite answer of whether they heated all the time or never heated their hydromorphone-CR injectates, which does not reflect the reality of injection behaviors. In other local literature, heating drugs has been shown to reduce bacterial load within cookers which contain hydromorphone-CR [12]. These findings have been translated into public health campaigns promoting “cooking one’s drugs” to reduce infectious complications of IVDU in London, Ontario [34]. It may be that our cases and our controls were both engaging in this behavior, but our sample size was insufficient to detect any difference.

Another hypothesized risk factor for IE was the site used for injection. Entrenched drug users tend to have thickened scar tissue from chronic injections in the same location; in many cases, this will be near the veins of the arm [35]. Furthermore, difficulty in accessing common sites may lead participants to inject in multiple sites for their hit, which can further increase the risk of infections given the multiple entry points for bacteria. Alternative sites of injection and IE likely reflects a greater difficulty in accessing safer sites, with alternate sites having a greater likelihood of contamination. Alternative injection sites may also be a surrogate marker for more venous damage from previous injections and thus entrenched drug use [29]. In particular, one study of PWID in the UK found the high-risk practice of injecting into the jugular vein was associated with the female gender and multiple body-site injections [29]. We found a significant difference between cases and controls with respect to the site of injection, which was driven by a higher frequency of cases using multiple sites. This was also seen in unadjusted logistic regression, where injection into multiple sites was associated with higher odds of IE (2.29; 95% CI 0.83, 6.07), albeit not statistically significant (*p* = 0.10). This effect was somewhat diminished when adjusting for sex and government-dispensed IDPE (OR 1.67; 95% CI 0.53, 4.97).

Our multivariable analyses did not include reuse of hydromorphone-CR for a second wash given the universality of this practise in our cohort and our sample size. A review of the literature had shown that conducting multiple washes of hydromorphone-CR injectates could also serve as a risk factor for IE. We did not find an increased likelihood of performing multiple washes with hydromorphone-CR (88% vs 74%). Our sample size was likely inadequate to identify these differences because the frequencies of both of these behaviours were much greater in both groups than expected. However, a companion study surveying PWID in London found that PWID with HIV were 22.12 (4.51 to 108.59) times more likely to share cookers, filters, or washes in their three-month recall period [14]. The high-risk practice of injecting prescription opioids from equipment that is reused multiple times is prevalent in our region and appears to be related to a high incidence of infectious complications including Hepatitis C and a very high incidence of IE [4, 36]. The trend towards increased use of crystal methamphetamine in controls may be suggestive that participants using less hydromorphone but who substitute with other agents, may be at lower risk of IE.

Homelessness and unstable housing have been associated with injecting in public spaces and other high-risk injection practices [37, 38]. However, in exploratory analysis, cases (PWID IE+) were not more likely to lack stable housing compared to controls. This is supported by Roy et al. [16], who found that unstable housing was not associated with conducting multiple washes (utilizing residual drug for multiple injections), which is often the preparatory method used to inject prescription opioids, such as hydromorphone-CR. We hypothesize that PWID using hydromorphone-CR, which is a more costly illicit substance, can be associated with stable housing, which is reflective of financial stability. Hydromorphone is one of the most expensive prescription opioids to purchase illicitly, costing Canadian $5.57/mg (US$4.28/mg) or Canadian $100.26 for an 18-mg capsule [39].

Interestingly, we found that our cases were more likely to have completed secondary or post-secondary education (61.3% cases vs 33.7% controls). In contrast, previous studies have linked the incompletion of education to illicit substance use [40]. Higher education likely is correlated with income and again may reflect greater accessibility to expensive prescription opiates such as hydromorphone-CR, which has properties that increase the risk of infections [12]. Our evaluation of the relationships between housing status and education with IE were exploratory and will require further studies to confirm.

## Limitations

A major limitation of this paper is that social and relationship factors which may put women at increased risk of IE were not explored in this survey. Limitations on the number of questions which could be asked in a single sitting led to this limitation, but it is important that these factors be extensively explored in subsequent studies. Further research should question whether people inject alone, use with a steady partner, are able to self-inject or rely on someone else most of the time, and whether they use with someone who controls their injection practices, such that they have no choice when receiving a used needle (and thus have a greater risk of infection).

Case–control study design may lead to recall biases with cases more likely to recall perceived hazardous behaviors. Our study reviewed cases admitted to or transferred (due to IE complexity) to the hospitals in the city of London. We did not capture cases admitted to regional or rural sites as other research has found poor harm reduction practices prevalent in rural settings [20]. Our controls were PWID accessing community resources as well as those presenting to outpatient clinics for other infectious complications. We did not capture PWID that do not seek medical or community supports, potentially missing controls who inject only at home, are not actively followed up for their addiction disorders or are of high socioeconomic status. These patients may therefore be less likely to perform high-risk behaviors. In particular, using the provincially distributed harm reduction materials may have been more common due to the controls having more access to these as many were attending addiction service centers.

Providing a monetary compensation for participation could have compromised the integrity of participant responses received; although, the relatively modest funds given in this study make this less likely. Finally, the relatively small sample size limited our ability to identify several hypothesized relationships. Further larger studies would be helpful.

## Conclusion

This study did not find an association between hydromorphone-CR use and infective endocarditis. The very high prevalence of hydromorphone-CR use in London possibly made our sample size too small to identify a significant association. We found being a female PWID and not using clean injection materials were risk factors for IE. Our work supports the necessity of harm reduction equipment distribution programs in reducing infectious complications among PWID. Further study of the potential social relationships as well as biological factors that may lead to a higher risk of IE in women are warranted.

## Abbreviations

IE: Infective endocarditis
PWID: People who inject drugs
IVDU: Intravenous drug use
Hydromorphone-CR: Hydromorphone controlled-release
IPDE: Injection drug preparation equipment
STROBE: Strengthening the Reporting of Observational Studies in Epidemiology
